# Combinatorial Antimicrobial Susceptibility Testing Enabled by Non-Contact Printing

**DOI:** 10.3390/mi11020142

**Published:** 2020-01-28

**Authors:** Adam S. Opalski, Artur Ruszczak, Yurii Promovych, Michał Horka, Ladislav Derzsi, Piotr Garstecki

**Affiliations:** Institute of Physical Chemistry of the Polish Academy of Sciences, Kasprzaka 44/52, 01-224 Warsaw, Poland

**Keywords:** antibiotic susceptibility test, non-contact printing, drug–drug interactions, antimicrobial resistance

## Abstract

We demonstrate the utility of non-contact printing to fabricate the mAST—an easy-to-operate, microwell-based microfluidic device for combinatorial antibiotic susceptibility testing (AST) in a point-of-care format. The wells are prefilled with antibiotics in any desired concentration and combination by non-contact printing (spotting). For the execution of the AST, the only requirements are the mAST device, the sample, and the incubation chamber. Bacteria proliferation can be continuously monitored by using an absorbance reader. We investigate the profile of resistance of two reference *Escherichia coli* strains, report the minimum inhibitory concentration (MIC) for single antibiotics, and assess drug–drug interactions in cocktails by using the Bliss independence model.

## 1. Introduction

In this paper, we present a method to perform high content biological assays in microwells that are prefilled by non-contact printing (spotting). Spotting allows for the deposition of well-defined amounts of active substances to arbitrarily chosen locations. Since bacteria show an alarmingly growing resistance to antibiotics, combinations of drugs are often used as a last-resort solution. Drug–drug interactions, however, need to be individually investigated for each patient, which calls for a simple, fast and affordable method. Here we demonstrate a proof-of-concept of a high-content combinatorial antibiotic susceptibility assay and identify the concentrations at which drugs interact. We use an easy-to-use and disposable cartridge that allows for the investigation of 1024 drug combinations in a single experiment with just two pipetting steps.

Bacterial infections are considered a serious risk for human health, e.g., each year around eight million cases of bacterial urinary tract infection are diagnosed in the United States alone [1]. Most bacterial infections can be successfully treated with antibiotics [2]. However, antimicrobial drug resistance, particularly last resort antibiotic-resistant strains, emerge at alarming rate [3,4,5]. Bacteria acquire resistance to drugs alarmingly faster than new antibacterial agents develop. Thus, new strategies for the fighting of bacterial resistance are sought-after.

Combinational treatment is one of the very limited options of fighting bacterial resistance to antimicrobials. Using cocktails of antibiotics might lead to an increase of the treatment efficacy [6,7,8,9,10,11]. This is why multidrug treatment are used not only to fight bacterial infections (e.g., urinary tract infections (UTI) [12] or tuberculosis [13]) but also HIV and cancer [14]. Research on drug–drug interaction is mostly focused on finding new, more effective ways to treat infectious diseases. Interactions between drugs can be categorized into three distinct types: non-interaction (or additivity), synergism, and antagonism. The mathematical definition of additivity has long been a subject of controversy. Recently, this definition has been widely accepted as the combination of the actions of the individual drugs and is considered as the baseline effect in the detection of drug–drug interactions. A synergistic effect occurs when the mixture of antibiotic shows a higher effect than a simple additivity and antagonism—when the effect of a combination of drugs is smaller than the sum of the actions of both drugs.

When used in combinations, drugs may interact with each other, which may lead to increased or decreased therapeutic effects. Changes in effects can be measured by monitoring bacterial proliferation or viability. Interactions between drugs can be described by using the Bliss independence (BI) model (also called the response multiplication model), which is one of the most commonly used approaches [10,15,16,17,18,19]. In the BI model, the assumption is that drugs do not interact with each other, and the result of a two-drug combinatorial treatment should be the multiplication of the drugs’ individual effects. Deviations from the expected values, δ, mean that drugs interacted with each other, either synergistically (if the effect of the drugs is higher than expected, δ > 0) or antagonistically (for an effect smaller than expected, δ < 0). Other known models that use different assumptions are the Loewe additivity [20], zero-interaction potency (ZIP) [21] or highest single agent (HSA) models [22]. There are freeware programs that are available that calculate drug–drug interactions, like SynergyFinder [19] or Combenefit [17].

Despite knowledge on drug–drug resistance being important to fighting drug-resistant infections, drug interaction studies are not commonly performed in clinical practice—mostly due to the high cost of labor. One of the limiting factors of running drug interaction studies is that they are specimen-specific—each of the bacterial strains is slightly different and may feature varying degrees of resistance to many antibiotics due to genetic differences [23]. This is why the introduction of simple-to-use tests that quantify the drug–drug interactions for a given clinical isolate would be greatly beneficial in diagnostics.

The antibiotic susceptibility test (AST) is conducted in order to determine whether a microorganism is susceptible or resistant to a drug or a combination of drugs. The results of the AST are values of minimum inhibitory concentration (MIC)—the lowest concentration of the drug that inhibits the proliferation of bacteria. ASTs can be carried out by using classical methods, such as broth dilution or agar diffusion methods [24]. However, they are often carried out manually, limiting their usefulness for high-content testing.

Current progress in microfabrication techniques has allowed for the development of high-content platforms for antibiotic susceptibility testing. One of the new approaches for carrying out ASTs is the employment of microfluidic devices with arrays of microwells, offering a higher density of sampling than conventional multitier plates (MTPs). Microwells are filled with drugs and bacteria, and their response is monitored and analyzed [25,26,27,28,29]. Such devices allow for the high-content investigation of single antibiotics, but they have limited use for combinatorial treatment schemes due to the problem of the delivery of well-defined amounts of drugs to the microwells. The delivery systems either rely on series of dilutions (allowing for only serial dilutions to be tested limits the assay’s dynamic range) or require a complex delivery system [30]. Droplet microfluidic systems, on the other hand, offer the potential to use a great number of combinations. Their drawback, however, is their need for a complicated experimental procedure and for a well-equipped laboratory and trained staff [10]. To reduce the time of the AST, a unique approach was recently demonstrated [31] in which single-cell bacteria are trapped in a long and narrow microchannel. Observing the growth rate of the bacteria allows for the phenotypic determination of their resistance against the supplied drugs. This method is very fast (under one hour with preparation), and it allows for a test that only needs a small sample size (few hundred bacteria), so it is not suitable to test polymicrobial infections or slow-growing bacteria species. Moreover, its sensitivity highly depends on which body fluid the sample is collected from, and its operation requires a well-equipped facility and skilled users.

Some researchers still employ pipetting robots that can handle multitudes of well plates; this is a well-established, time-consuming, and expensive method [23]. Classical ASTs are labor intensive, and state of the art systems (e.g., VITEK^®^2 (bioMérieux SA, NC, USA) and Phoenix^®^ (Becton Dickinson, NJ, USA)) offer categorical information about susceptibility by referencing the observed growth to breakpoints [32].

The detection in an AST can be carried out simply via the naked eye, either by measuring the inhibition zone with a ruler [24] or by monitoring the colorimetric change of an indicator dye due to the processes occurring in the sample (e.g., resorufin to resazurin conversion [33]). More sophisticated but also more precise methods include a readout of fluorescence intensity (fluorescently labelled bacteria or the use of dye) [10] and the monitoring of the optical density of the sample [34].

One of the commonly used tools for high-content biological tests are plates that feature arrays of small wells, micrometer to millimeter in size [35]. Microwell arrays are a popular choice because they are usually compatible with widespread optical readout methods, such as plate readers or fluorescent microscopes [36]. In each of the independent wells, a different experiment can be carried out, depending on the content of the well. The delivery of the tested compounds to the wells can be a limiting factor of an assay’s applicability. The dispensing number of various solutions into numerous wells requires either complex equipment (such as 22 valves for filling 12 wells [30]) or limits the number and dynamic range of the investigated compounds (serial-dilution based gradients of two solutes in 600 wells [37]).

Non-contact inkjet printing (spotting) is a reliable method of the delivery of small volumes of liquids to pre-defined locations [38,39,40,41]. Spotting features a high accuracy and precision of sample delivery, both of which are characteristics of a great tool for delivery of reagents into microwells [42]. Great control over the spotted volumes allows for the precise deposition of the desired amount of substance (e.g., drug) at predetermined spatial coordinates (spots). Each spot is printed individually, which allows for the creation of arrays of substances in arbitrary locations, with well-defined concentrations. Deposited solutions dry quickly and can be later dissolved [28]. A spotting machine, because of the cost and need for trained staff, is usually found in centralized spotting facilities. However, plates with dried substances can be easily vacuum packed, shipped and stored [28]. If the assay that uses the spotted plates is easy in execution and robust, the centralized preparation of antibiotic assays increases the availability of said assay.

Here, we show an easy-to-use method for the determination of MIC and for assessing drug–drug interactions. Operations are carried out in a microfluidic device comprised of microwells with an antibiotic deposited by non-contact printing. The device, called the mAST (microwell AST) features 1024 submicroliter wells. This number of wells allows us to perform 16 checkerboard-experiments simultaneously, each presenting a full eight by eight matrix of combinations of concentrations of two antibiotics. In our demonstration, we show four pairs of antibiotics, each repeated four times in a single experiment. This allowed for a high-resolution readout in a test powered by two filling steps. The mAST can be used in any laboratory with a vacuum pump, an incubator, and a laminar chamber. In our experiments, which monitored the optical density of the wells, we tracked the growth curves of bacteria in each well. The presented AST is also compatible with simpler reading procedures, like the use of pH-sensitive dyes and the observation of changes of the well color [27,30] (see Electronic Supplementary Information, ESI). In short, if a medium contains dye and a source of food for bacteria that can be converted into pH-changing agents such as CO_2_, then, after incubation, the wells containing proliferating bacteria should change the pH and the color of the dye. This could be a basis for developing a cell-phone based app for the readout of the mAST. Another possible technical development could be fabricating the mAST with wells placed analogously to well-plates (384 or 1536 wells per plate) in order to use commercially available plate readers for readout.

## 2. Materials and Methods

**Figure 1:**
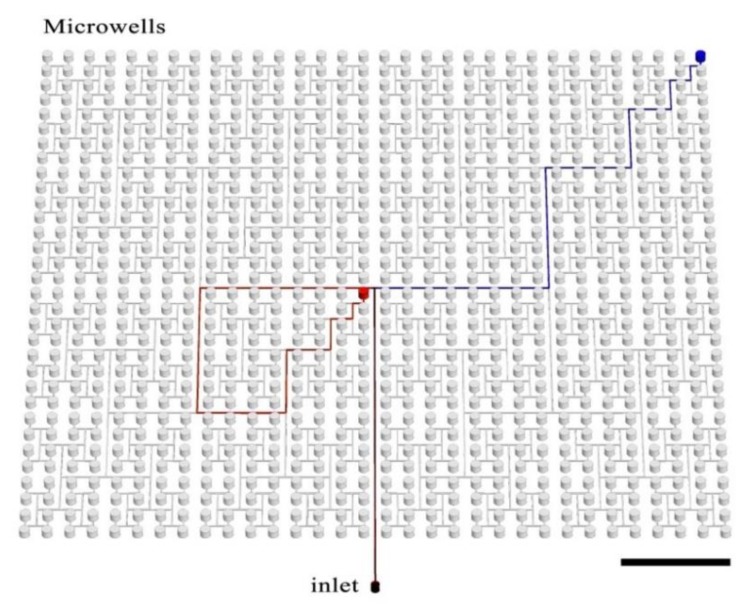
Schematic architecture of the fractal microwell array. Grid of microwells, which are connected by a network of branched microchannels. We highlight the two paths the fluid takes to fill two microwells with colored lines in order to show that all paths to all microwells are hydraulically equivalent. The scale bar is 1 cm.

The presented device comprises 1024 microwells, each of diameter 1 mm and volume 0.59 μL. The total device volume is ~628 μL and consists of the wells (603 μL) and the delivery channel network (25 μL). Wells are arrayed in a single polydimethylsiloxane (PDMS) plate in 32 rows and 32 columns that are spaced uniformly on a square grid. Each microwell is connected with the inlet by a branched microchannel (see Figure 1), as described previously by Zhu and coworkers [43]. Antibiotics are deposited in wells by non-contact printing [44].

**Figure 2:**
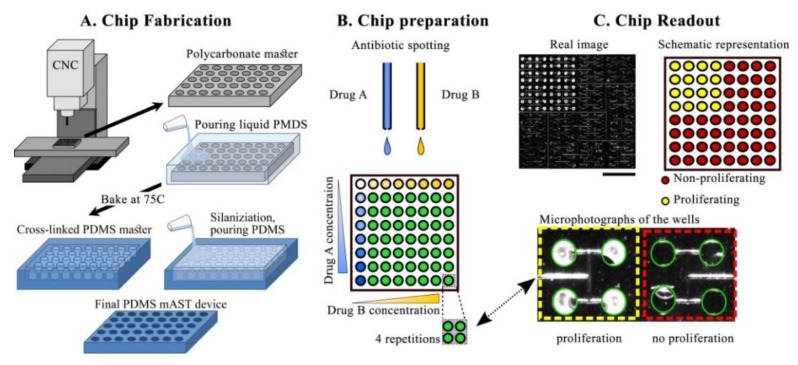
Preparation and readout of a high-content microwell antibiotic susceptibility test (AST) (mAST). (**A**) Fabrication of the mAST device. (**B**) Preparation of the chip by spotting gradients of antibiotics, in 4 repetitions each. (**C**) Readout of the mAST after incubation. **C Top Left**: image of the mAST with brighter microwells (proliferating bacteria) and darker (unchanged) wells. Scale bar is 1 cm. **C Top Right**: binary representation in which wells the bacteria proliferated. **C Bottom**: microphotograph of part of the mAST with 4 wells with proliferating bacteria (in the left, bright) and 4 wells without bacteria proliferation (in the right, dark). Well contours are outlined in green to guide the eye. The small areas of different color within microwells are trapped gas bubbles of different refractive indexes than the culture medium. Width of the well is 1 mm, which can serve as scale bar.

A scheme of the mAST fabrication is shown in Figure 2A. A microfluidic device was designed in MasterCAM 2018 (MasterCam, CNC software Inc., Tolland, CT, U.S.A.USA). The delivery microchannel was CNC-milled (MSG4025, Ergwind, Gdańsk, Poland) in a 0.75 mm-thick polycarbonate plate. Then, the microwells were drilled through the same plate, which was then thermally bonded to 5 mm-thick polycarbonate plate, as described before [45] (both plates were made of Macrolon^®^ GP, Covestro, Leverkusen, Germany). A polycarbonate chip served as a positive mold from which a negative replica was cast in PDMS (Sylgard, Dow Corning, Midland, MI, USA), by pouring over a 10:1 PDMS:cross-linker solution, degassing, and baking at 75 °C overnight. The PDMS negative surface was then protected by silanization (3 h under 10 mbar with vapors of tridecafluoro-(1,1,2,2-tetrahydrooctyl)-1-trichlorosilane (Alfa Aesar, Thermo Fisher GmbH, Kandel, Germany) and served as a master mold for the final PDMS devices. The proper devices were obtained by filling the negative mold with the degassed 10:1 PDMS:cross-linker mixture, degassing, and baking overnight at 75 °C. The replicated PDMS device was peeled off, an inlet hole was punched through it, and, finally, the device was thoroughly washed and dried with pressurized air prior to deposition of the antibiotics.

Stock solutions of antibiotics were spotted to the wells by using non-contact printer (spotter) SciFLEXARRAYER S3 (Scienion, Berlin, Germany) and a hydrophobically-coated piezo dispense capillary (PDC 70, modification type 2, Scienion, Berlin, Germany). The AST workflow, including the spotting process, is illustrated in Figure 2. After spotting, the solvent evaporated, and dried deposits of antibiotics were left in predefined positions. The dry chips were sealed with adhesive (AB-580 from Thermo Fisher Scientific, Waltham, MA, USA).

**Figure 3:**
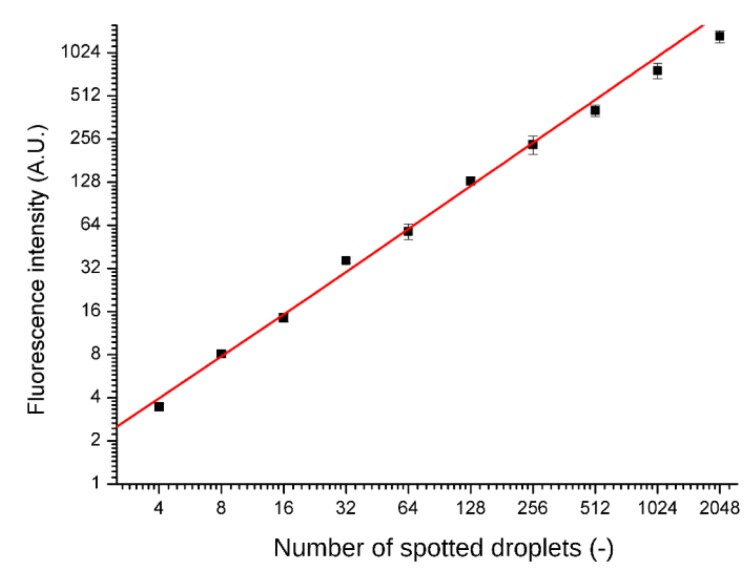
A relationship between the spotted amount of dye and normalized fluorescence (each point is an average from 4 wells). The fluorescent measurement was performed after 6 h of filling the chip, i.e., after a typical culture time. Red line is a linear fit of the data points (R^2^ = 0.96779).

Spotting accuracy and precision was tested by printing fluorescent dye (rhodamine 110 chloride, Sigma Aldrich, Darmstadt, Germany) into the microwells. After drying the solution and sealing the chip, the dye was re-suspended in the supplied distilled water and separated by fluorinated oil FC-40. Fluorescence intensities were read out with a confocal microscope after overnight incubation (A1R, Nikon, Minato, Tokyo, Japan) and are presented in Figure 3. No cross-contamination between the wells was observed.

The inlet of the PDMS device with deposited antibiotic concentrations was sealed, and the device was sterilized by ultraviolet irradiation (UV, 30 min). UV is known to affect the activity of some compounds, e.g., antibiotic gentamycin activity has been shown to be greatly reduced if the plate is sterilized with UV, and this was the reason for not including this drug in the tested panel. All of the tested drug activities are the same for the irradiated and non-irradiated plates. The sterile chip was degassed (10 mbar for at least 30 min). We used the chip immediately after degassing. Others have demonstrated that it is possible to store such pre-vacuumed chips in food-grade vacuum sealed bags [28]. After removing the inlet-covering foil, a pipette tip with a bacteria inoculum sample was placed in the inlet. The amount of the sample (0.6 mL) was calculated so that each well was fully filled without leaving sample leftovers in the connecting channels. The degassed PDMS pulled air in, drawing the sample into the microwells and filling them completely. The system was dead end because the pockets of air were absorbed by the degassed PDMS. Before the sample was fully aspirated into microwells and air started invading the microchannel (ca. 5 min), fluorocarbon oil FC-40 (3M, Maplewood, MN, USA) was added to the tip at the inlet. The inert fluorinated oil filled the channels just after the sample. Once the sample was distributed evenly among the microwells, thus filling them, and the oil that followed it reached the brink of microwells. Hence, the oil separated wells from each other and did not hinder the bacteria growth due to biocompatibility and a high air content [46]. Bacteria, however, always stay in the aqueous phase [47]. In the wells, antibiotics dissolved in the aqueous sample, and the desired concentrations of the antimicrobial agents were reached. The total filling time depends on the process of degassing (time and vacuum level)—for the presented condition, it took under 10 min.

In our experiments, we used two strains of *Escherichia coli.*: (i) a reference strain without additional drug resistance (ATCC 25922, called ‘non-producer’ in this work) and (ii) a strain producing beta-lactamase (ATCC 35218, called ‘beta-lactamase producer’ in this work). Those strains are standards that are used commonly in the AST. *E. coli* ATCC 35218 is recommended as the quality control organism for the β-lactam–β-lactamase inhibitor agents. Both strains were plated on an LB agar medium (Sigma Aldrich, Darmstadt, Germany) and incubated overnight at 30 °C. After incubation, we picked a couple of colonies that were diluted in 0.95% NaCl and adjusted to 0.5 McFarland with the use of a densitometer (Biosan, Józefów, Poland). Such prepared inoculums were diluted 200 times in a Mueller Hinton medium (Biocorp, Warsaw, Poland) to obtain final concentration of 5 × 10^5^ CFU/mL. All used antibiotics were purchased from Sigma Aldrich (Darmstadt, Germany). Stocks of antibiotics were dissolved in (i) sterile water (ampicillin (AMP), piperacillin (PIP), imipenem (IMI), spectinomicin (SPX), and streptomycin (STR)); (ii) 0.1 M NaOH (ciprofloxacin (CPR)); (iii) DMSO (trimetoprim (TMP)); and (iv) ethanol (chloramphenicol (CHL)). Stock solutions of antibiotics were diluted in sterile distilled H_2_O and transferred into a 384-well microtiter plate (Scienion, Berlin, Germany), and from there, they were printed into the chips by the spotter.

**Figure 4:**
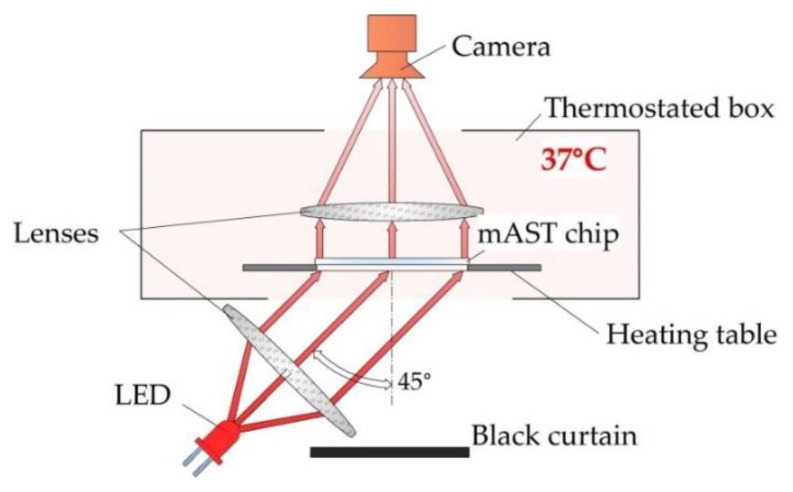
Schematic depiction of the detection setup.

Bacteria-laden chips were incubated at 37 °C in a homemade setup, where the optical density of the liquid in the microwells was measured overnight. The detection setup is schematically shown in Figure 4.

We used label-free detection, i.e., we detected and measured the intensity of light scattered on bacteria with a camera (MD120MU-SY, Ximea, Münster, Germany) without adding any chemical markers for growth. As a light source, we used collimated light beam from an LED (wavelength 640 nm), illuminating the chip from the bottom at 45°. The temperature inside the optical density (OD) reader was controlled by heating the table on which the microfluidic device with the sample was placed, and, if needed, additional air heating from the sides of the device was used. All three heat sources were PID-controlled and provided a homogeneous temperature distribution inside of the device. Images of the of the mAST chip were collected in 5-min. intervals. From each image, data on the light scattering were extracted for each well and plotted (see ESI for details).

## 3. Results

**Figure 5:**
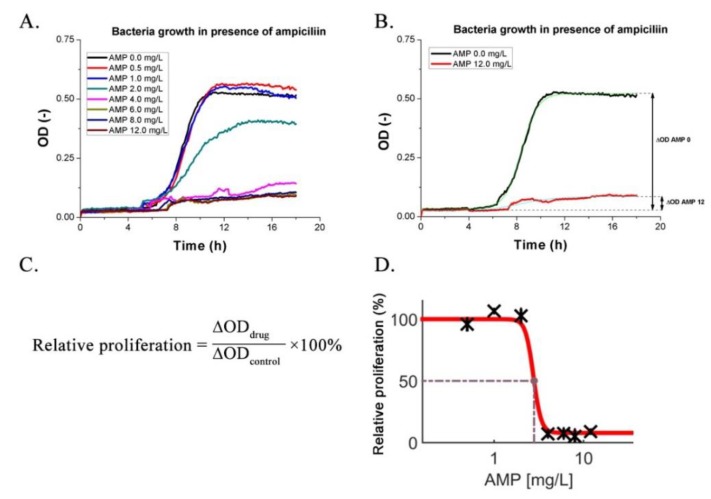
**Top:** Growth curves of bacteria in presence of ampicillin at various concentrations. MIC (minimum inhibitory concentration) determination is a choice of the lowest concentration that inhibits growth (MIC = 4.0 mg/L). (**A**). Each growth curve was fitted with a sigmoidal dose–response function (**B**). The ΔOD (change in optical density) is a difference between the baseline and the highest recorded response inferred from the fit. **Bottom:** Calculation of the drug impact on the bacteria population. Proliferation was calculated as the ratio of the signal from the investigated well to a control well with bacteria without a drug (**C**). A dose–response plot is shown in the right without first point, which is control at concentration zero (**D**).

For single antibiotics, the MIC was determined as the lowest drug concentration for which the bacteria proliferation dropped to a low and constant level (see Figure 5D) [48]. As an example, in Figure 5, we present a way to quantify the MIC. Firstly, the bacteria were cultured in the wells of the mAST after being prespotted with a drug. The light scattering (OD) of each well was continuously monitored (see Figure 5A). A sigmoidal curve was fitted to each growth curve, and a change in OD during the experiment was established (ΔOD, see Figure 5B). By comparing the ΔOD of the investigated microwell to the ΔOD from the control well, we obtained a measure of the drug effect on the bacteria, which was expressed as the proliferation factor (see Figure 5C). Changes of bacteria proliferation rates were plotted against the drug concentrations by using a dose–response model, and the MIC was read as the first point on the lower arm of the curve (Figure 5D). For calculations and plotting, we employed Combenefit [17], we observed some slight increases of the OD signal regardless of the concentration of the antibiotics. We believe that this was an artefact due to the porous character of the PDMS, which, in time, absorbed some small amount of the antibiotic solution and changed background noise (weak optical signal reflected from the walls of the wells—see Figure S4).

To establish the MIC for single antibiotics, we spotted each substance to subsequent wells prior to filling the wells with the bacteria solution. After the incubation at 37 °C for 18 h, the MIC was determined. Each series was repeated four times in the neighboring wells. A control experiment was carried out in the 96-well plate by using serial dilution method. We used two strains of *E. coli.*: (i) ATCC 25922 and (ii) ATCC 35218, the latter of which is a beta-lactamase producer. The results are summarized in Table 1.

The MIC values were determined with the mAST method within essential agreement (i.e., within a one two-fold dilution) to the MICs that were obtained from the reference plating test (one-fold dilution differences). Our results were within in EUCAST susceptibility reference ranges [49], so our system was accurate in the determination of the bacteria’s susceptibility to drugs. This is crucial, as the false identification of a resistant pathogen as a susceptible one is extremely dangerous and could have a huge impact on the successful treatment and health of the patient.

**Figure 6:**
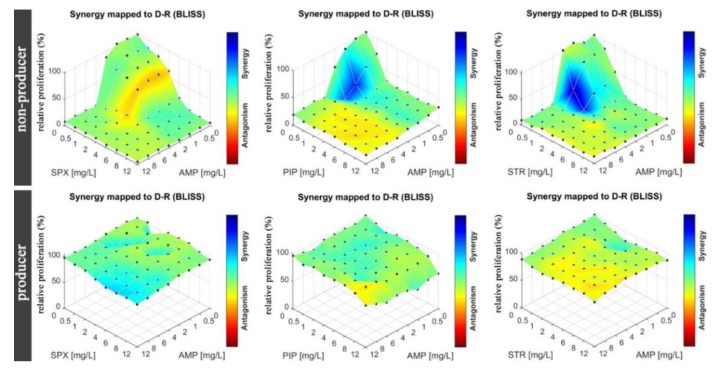
Bacteria proliferation as a function of two drug concentrations, where one of the drugs is ampicillin. Bliss scores overlapped over the proliferation map (color-coding). (**Top**) bacteria that do not produce beta-lactamase. (**Bottom**) Beta-lactamase producing strain. Each row represents results from a single mAST containing 4 experiment repetitions.

In the next step, we tested drug–drug interactions for the same strains of *E. Coli* as in the MIC determination. Eight by eight checkerboards of pairs of drugs were deposited into the mAST (drug concentrations: 0–12 mg/L for AMP, PIP, SPX, and STR). Inoculum of bacteria were supplied to the microwells and then incubated overnight while their viability/growth curve was monitored by continuous light scattering readout. The proliferation of the bacteria was assessed, and the drug–drug interactions were calculated and are graphically depicted in Figure 6.

**Figure 7:**
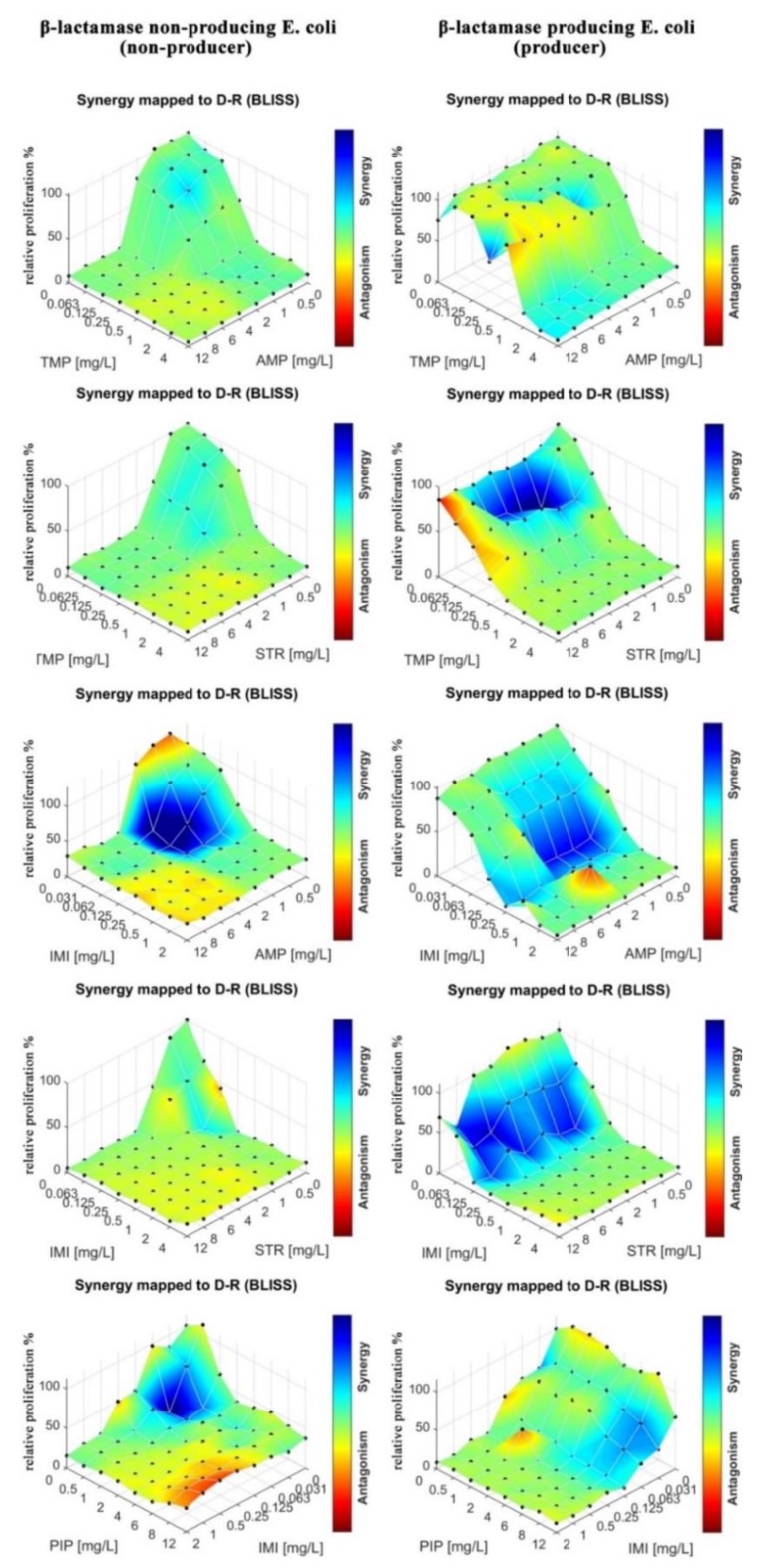
Relative proliferation of bacteria calculated as the ratio of the signal from the investigated well to the control well with bacteria without a drug; this was plated as a function of two drug concentrations in various combinations. Synergy scores overlapped over the proliferation map (blue–red color coding). Bacteria that do not produce beta-lactamase (non-producer) were placed next to the bacteria that do produce beta-lactamase (producer) for the same pair of tested antibiotics. Each map represents the results from a single mAST containing 4 experiment repetitions. More information can be found in the ESI.

To assess drug–drug interactions, we used the Bliss independence model, as explained in the introduction, ESI and literature [10,15,16,17,18,19]. We measured deviations of the measured effect of the drug cocktail from the expected value of the two drugs that acted independently. Deviations from the expected values, δ, were a sign that the drugs interacted synergistically (δ > 0) or antagonistically (δ < 0). For the analysis of our experiments, we calculated δ by using algorithms that were included in the COMBENEFIT software (v. 2.0.2, Cancer Research UK Cambridge Institute, Cambridge, England) [17], and we present the results in the form of a color map (a color bar next to plots in Figure 6 and Figure 7). A red color means strong antagonism, blue indicates that the drugs acted synergistically.

We observed that in the case of the strain that did not produce beta-lactamase, all tested antibiotics (AMP, PIP, STR, and SPX) were effective as both single agents and in cocktails. Some combinations proved to be slightly more effective due to synergistic effects (AMP at around 1 mg/L and STR or PIP at 0.5–1 mg/L acted stronger together rather than separately). In the case of beta-lactamase producer bacteria, we did not observe an effect of the tested drugs, either alone or in combinations.

In the next step, we tested drug–drug interactions for different drug combinations. We tested AMP, IMI, PIP, TMP, and STR in combinations. In contrast to previous experiment, not every tested combination contained ampicillin. As stated previously, the mAST contained spotted eight by eight checkerboards of drug pairs. The growth of the bacteria in wells was monitored, and interaction maps between the drugs are plotted in Figure 7.

We observed mostly additive and synergistic interactions between the drugs in the tested combinations of antibiotics that represented different antibiotic groups, and we only observed sparse regions of antagonistic interactions. Most notably, IMI and TMP enhanced the potency of the other drugs against the beta-lactamase producing *E. coli.* strain. IMI is a last resort antibiotic that is known for its adverse effects on patients [50], and it should be used only when all other antibiotics fail to offer an effective treatment. Our results suggest that TMP is an effective alternative to IMI, with a similarly pronounced synergy in the tested combinations. A particularly interesting combination is STR–TMP when used against the beta-lactamase producing *E. coli.* Though we previously found this strain to be resistant to aminoglycoside STR (see Table 1 and Figure 6), we found here that for a particular dosing range (STR 1–6 mg/L, TMP 0.0625–0.125 mg/L), there was a synergistic interaction between TMP and STR where the bacterial proliferation was severely limited.

## 4. Discussion

We presented a simple and disposable microfluidic device for antibiotic susceptibility tests (mASTs). The active substances are pre-deposited by non-contact printing, and the devices are operated by a conventional air displacement pipette to perform an AST in a couple of steps with minimal hands-on time. As a proof-of-concept, we tested two bacterial strains for six drugs, individually and in combinations.

When testing single antibiotics (AMP, IMI, PIP, SPX, STR, and TMP) we identified two groups: drugs that were effective against both strains (IMI and TMP) and drugs that were only effective against a strain that did not produce beta-lactamase (AMP, PIP, SPX, and STR). The obtained MIC values comply with the EUCAST guidelines for susceptibility determination for one resistant and one susceptible strain [49]. When testing multidrug combinations, we counted on the drug–drug interactions to improve the efficiency of antibiotic treatment. Our expectations were met, as beta-lactam AMP and aminoglycoside STR, which had previously been found to be ineffective against beta-lactamase producing *E. coli.*, showed activity when paired with IMI or TMP.

IMI is a carbapenem antibiotic that is known for a high degree of resistance to degradation by beta-lactamases. IMI works by inhibiting bacterial cell wall integrity and synthesis; thus, when it is used in combination, it may assist other antibiotics to permeate the cell wall. However, IMI is associated with an increased risk of seizures [50], and it should be used only in the most severe circumstances as a last-resort antibiotic [51].

In our experiments, TMP showed a similar efficacy to IMI when combined with other antibiotics. TMP cannot be decomposed by beta-lactamase, which is why the drug is effective against the beta-lactamase producing *E. coli.* strain. TMP works by blocking the folate metabolism of the bacteria, and it is commonly used in synergic combination with sulfamethoxazole (SMX) to treat a variety of bacterial infections [52]. In such a combination, TMP and SXM inhibit sequential steps in the same biosynthetic pathway of tetrahydrofolate, but this generally accepted mechanism has recently been reappraised [53]. The synergistic interaction of STR–TMP that was observed in our test was unclear because the two drugs inhibited different actions, apparently without connection. STR is a protein synthesis inhibitor and a bactericidal antibiotic. It works by binding to the 30S ribosomal subunit of the bacterial ribosome, resulting in codon misreading and, eventually, the inhibition of protein synthesis; however, the ultimate bactericidal action of STR is yet not fully understood. Moreover, in contrast to TMP–SMX, the interaction of TMP–STR is poorly studied, and the use of this antibiotic combination against bacterial infections has rarely been reported [54]. Our results may therefore inspire further investigations of its synergic mechanism and therapeutic use.

For the readout, we used a custom-built optical detector that allowed for label-free detection based on the light that was scattered on the proliferating bacteria. However, one could use other methods for readouts, such as absorption spectroscopy or smartphone-based detection with 3D printing-suitable frames and holders for optical waveguides or for smartphones [55,56]. Even a classical plate reader could be employed after proper calibration if one could fabricate the mAST by placing the microwells on a grid of 384 or 1536-well microtiter plate. While we did not test all these possibilities, we have demonstrated a simple colorimetric assay (Figure S4) by using a pH indicator of the bacterial metabolism that can be detected with a naked eye.

## 5. Conclusions

Here, we presented high-content biological assay that was prepared by non-contact printing (spotting). The proof-of-concept experiment we chose was antibiotic susceptibility testing, as growing resistance to antibiotics is a global health hazard. We were able to obtain information on bacteria susceptibility and resistance (by measuring minimal inhibitory concentration) and to quantify the interaction profiles of six antibiotics by using the Bliss independence model. The presented method and device are promising tools for testing clinical isolates for drug resistance in hospitals in complement to existing methods.

The method that is presented in this paper features numerous advantages. Firstly, the AST can be performed in a label-free fashion with multiple replicates during a single run. Secondly, the operation of the device requires only two pipetting steps, which makes its use less prohibitive than complex biochemical assays. Lastly, a large number of compartments make the system suitable for the high-content profiling of strains’ drug resistance.

The drug resistance of bacteria that produce drug-decomposing enzymes, such as beta-lactamases, is a serious problem and might be a source of persistent infections that are difficult to eradicate. Using a combination of antibiotics if a single drug alone does not work against a pathogen is an effective method to combat resistant strains. We demonstrated that our device can easily identify such drug combinations on a large scale, providing valuable information to select effective treatment strategies.

It is important to note that there are commercially available systems with disposable cartridges for performing an AST, such as VITEK2 and Phoenix. They categorize multiple antibiotics into the resistant (R), susceptible (S), or between the two (I) classes. These systems, however, do not allow for automated studies of the efficacy of antibiotic combinations. Our system is complementary to the state of the art systems, as it allows for the study of drug–drug interactions.

## Figures and Tables

**Table 1:** MIC values for the two tested strains measured in the mAST device and via standard microdilution in microtiter plate (MTP) and compared to EUCAST breakpoints for susceptible (S) and resistant (R) strains by color coding: values for are green for susceptibility red for resistance, and grey for no data. Dose–response curves for tested drugs can be found in the ESI.

Drug	MIC Reference mAST [mg/L]	MIC Reference MTP [mg/L]	MIC Resistant mAST [mg/L]	MIC Resistant MTP [mg/L]	EUCAST [49] SR [mg/L]	
AMP	4	6	R	R	<8	>8
PIP	2	1.5	R	R	8	16
IMI	0.25	0.1	0.25	0.1	2	8
TMP	1	0.4	1	1.6	2	4
STR	2	3	>12 (R)	250 (R)	No data	No data
SPX	6	6	>12 (R)	>250 (R)	No data	No data

## Supplementary Materials

The following are available online at https://www.mdpi.com/2072-666X/11/2/142/s1, Figure S1: Image analysis methods: mask and regions of interest (ROIs) for well detection. Figure S2: Image analysis methods: affinity transformation. Figure S3: Image analysis methods: extraction of signal from ROIs. Figure S4: Images of the mAST during incubation at varying time. Figure S5: Bliss independence model. Figure S6: Synergy scores for drug–drug combinations containing imipenem for the strain producing beta-lactamase. Figure S7: mAST readout using pH indicator. Figure S8: Dose–response plots of drugs against reference an resistant strains. Figure S9: Readout variability for 4 repetitions.
