# Supplementary material for: Combinatorial Antimicrobial Susceptibility Testing Enabled by Non-Contact Printing
Source: Micromachines (Basel). 2020 Jan 28;11(2):142. doi: 10.3390/mi11020142 (PMC7074582; doi:10.3390/mi11020142)
Supplement: Supplementary file 1 [file micromachines-11-00142-s001.pdf]

micromachines

Electronic Supplementary Information: Combinatorial Antimicrobial Susceptibility Testing Enabled by Non-Contact Printing

Adam S. Opalski, Artur Ruszczak, Yurii Promovych, Michał Horka, Ladislav Derzsi * and Piotr Garstecki *

Institute of Physical Chemistry of the Polish Academy of Sciences, Kasprzaka 44/52, 01-224 Warsaw, Poland
* Correspondences: lderzsi@ichf.edu.pl (L.D.); pgarst@ichf.edu.pl (P.G.); Tel.: +48 (22) 343 22 33 (L.D. & P.G.).

Received: 3 December 2019; Accepted: 23 January 2020; Published: date

Data acquisition and processing - growth curves readout and fitting

To extract information on light scattering (and consequently bacterial growth), we:

- select and index microwells in the image (regions of interests, ROI). For this we used affine transformation of the prepared table of the coordinates of the wells;
- compute mean light intensity within ROIs to get the intensity of the wells;
- obtain growth curves and parametrize for them the sigmoid based functions;
- classify each well as growing/non-growing based on the sigmoidal function. For this stage we apply two-layer perceptron based applied neuron network with the back propagation error learning algorithm.

We extracted the information on the change of the absorbance using Labview® (National Instruments, Austin, TX, USA) script. Another Labview script plotted the growth curves and assessed the change of OD of the wells by the difference between arms of sigmoidal function. Viability of the bacteria in each well was calculated as percent of the control well (without drug). Surface plots of viability and interactions were prepared in COMBENEFIT [1] and Matlab® 2018b (Mathworks, Natick, MA, USA).

Data analysis

We processed images obtained during the incubation of the mAST as follow. Firstly, we detected the microwells by image analysis and then plotted the change of signal intensity in time for each of the microwells. The change of intensity of light refracted in the microwell corresponded to number of light-scattering objects in the microwell, i.e., bacteria.

Microwell Detection by Image Processing

To detect a microwell in the image we prepared a map of positions of the microwells in the mAST—a mask (Figure S1a). The mask contains information on spatial coordinates of rectangular regions of interests (ROIs) containing a single well each.

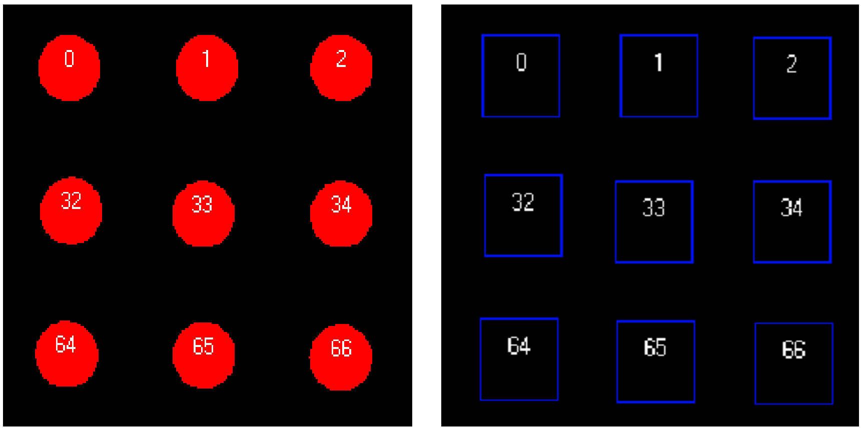

(**a**) (**b**)

Figure S1. Fragment of a mask with numbered ROIs (**a**), microwells detected in the ROIs (**b**).

To account for the chip size variations—due to PDMS elasticity and manual mAST preparation—we applied affinity transformation to the images. This allowed us to localize the ROIs containing microwells (Figure S2a). Coupling of the mask of microwells and the actual image is realized by aligning edges of mask to the image and affinity transforming the image. The procedure resulted in overlaying ROIs over the image, with each ROI containing a single microwell. (Figure S1b).

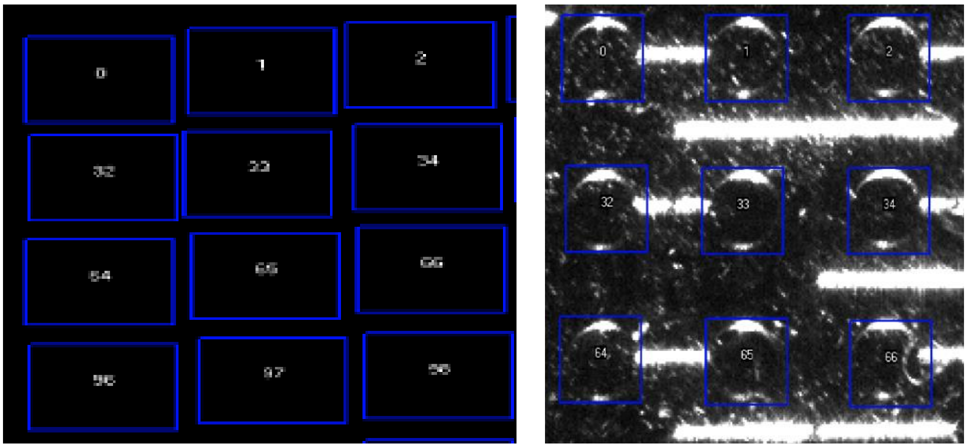

(**a**) (**b**)

Figure S2. Affinity transformation of mask (**a**) and resulting detected microwells (**b**).

We detected microwell in the ROI by analysis of 5 acquired images. The procedure is as follows: glow intensity of pixels from 5 last collected images is analysed. Then, saturated pixels were excluded from analysis, reducing dispersion of the growth curves and we excluded most of the artifacts coming from the light scattering on the edges of the microchannels and mAST.

There are two ways to identify area within ROI that is occupied by the microwell. First way relies on setting glow intensity threshold. If a difference in glow intensity between the value from the first image and average from the last 5 images is greater than the intensity threshold, then this pixel is considered as located within the microwell. The pixel glow intensity changed within the interval (0, 1) and the intensity threshold value was set to 0.2. The other way of microwell detection is by estimating the of dispersion of the glow intensity of pixels of the five last images. Dispersion of the glow intensity of the same pixel on images collected at the different times is significantly larger for pixels that corresponds to microwells and channels, than of the background. The threshold level for identifying pixel as belonging to a microwell or channel was 0.707 (one over square root of two) of the maximal dispersion of the glow intensity of pixels in the acquired images (Figure 4).

For microwells containing high bacteria concentration (light-scattering objects) both ways allowed for acurate detection of the microwell position in the ROI. However, microwells that are occupied by only a small number of bacteria (e.g., wells with high drug concentration and non-proliferating bacteria) are not detected properly when the threshold intensity method is used. Therefore, only the dispersion analysis was used throughout the analysis of the experimental data.

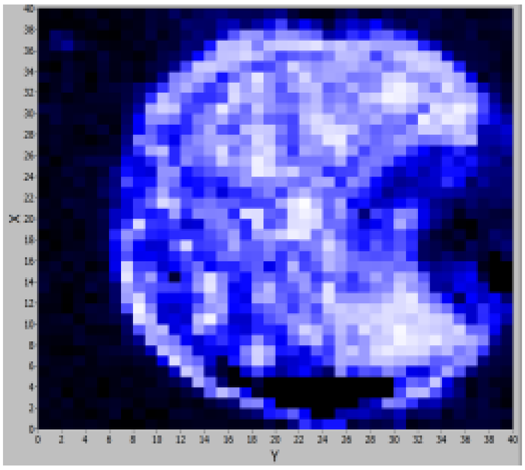

(**a**)

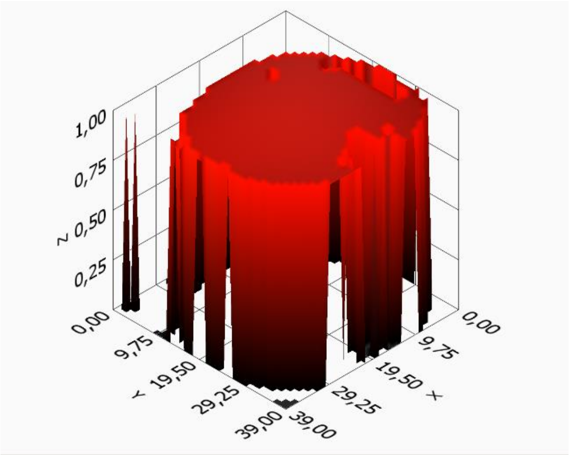

(**b**)

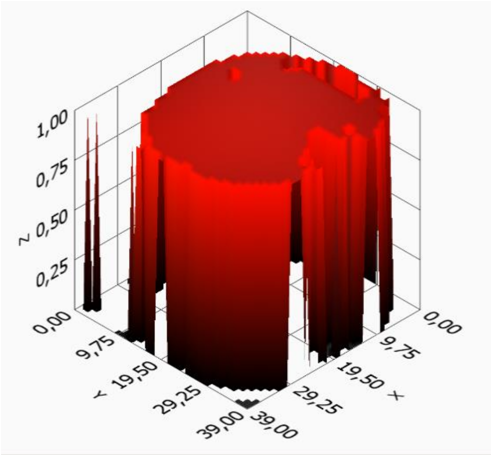

(**c**)

Figure S3. ROI of one of the wells without saturated pixels (**a**) and binary masks of the microwell region selected by glow intensity (**b**) and by dispersion of glow intensity (**c**).

Plotting Growth Curves

Pixel intensities of the microwell in a single image are averaged and constitute a single point on the growth curve for each microwell. All the artifacts coming from possible uneven mAST illumination, lights reflected from the walls of the wells, errors of the optical system and non-identical starting conditions are removed by arbitrary assigning the signal from timepoint = 0 the value intensity = 0.

We note, that very high concentration of antibiotics may also contribute to increased OD signal. In one of our experiments we treated E. coli treated with a potent antibiotic, ciprofloxacin (MIC ~ 3 µg/L). The highest tested concentration was 30 times of the MIC (100 µg/L) and yielded slightly higher OD value (Y-axis) than e.g. for the 3x MIC value. Nevertheless, removing the background noise at time t = 0 together with removing saturated pixels allows reduce the image artifacts influence from the gas bubbles that can form in the microwells during the experiments (See Figure S4).

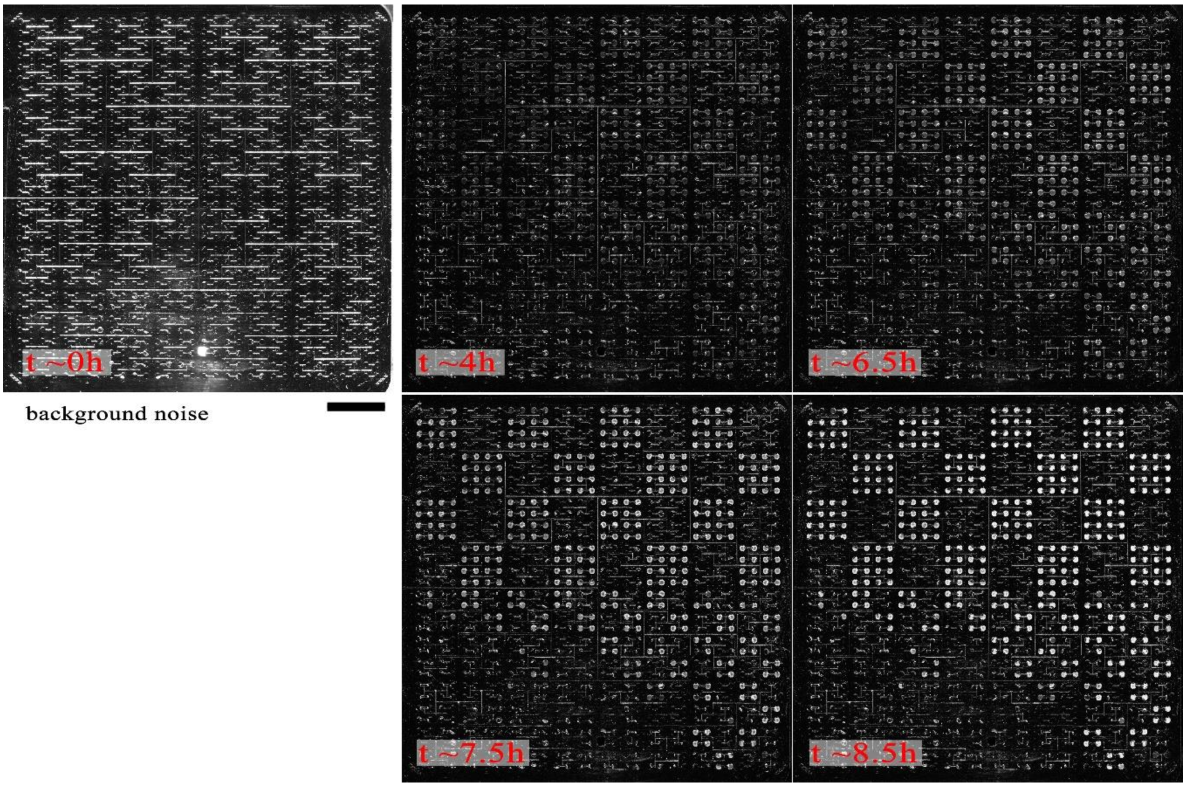

Figure S4. Images of the mAST during incubation at varying time. High concentration of ciprofloxacin (>10 MIC, 1 mg/L) was spotted in the checkerboard pattern. In areas where bacteria divide (no drug), the wells scatter light and appear brighter. Snapshot at t = 0 h shows the light reflected from the channel walls, generating static background noise. While this is excluded from the image analysis, for increase the visibility we extracted this background noise from time-sequence images (t ≈ 4–8.5 h). Bright spot in the bottom of all pictures is a refraction of light at the lens, which does not influence the readout (constant value, can be subtracted from the signal).

Combenefit for Measurement of Drug-Drug Interactions

For interaction quantification we employed Combenefit [1], a simple to use freeware program that plotted the drug dose-response graphs (see Figure 5D), assessed the drug-drug interactions (calculated δ score) and the statistical significance of the results (p-value) and plotted the 3D map of the bacteria proliferation and drug interactions (δ mapped over the map of the proliferation of bacteria for various drug doses)—as presented in Figure S5.

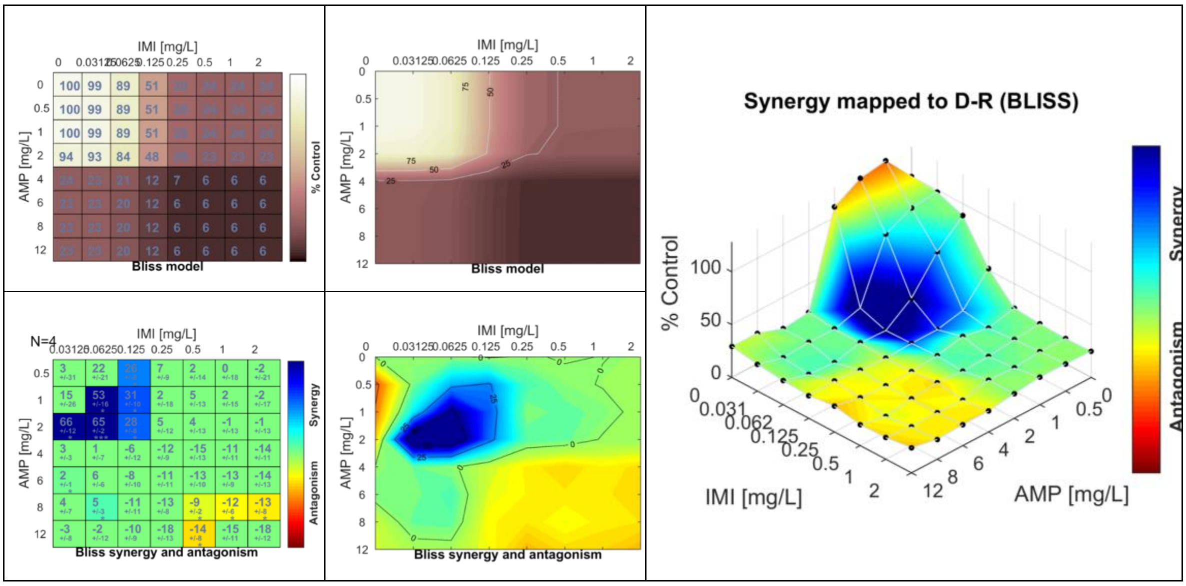

Figure S5. Bliss Independence model. **Top**: matrix (**left**) and isobologram (**right**) of expected viability scores for non-interacting drugs. **Bottom**: Deviations from the expected scores (δ) presented as matrix (**left**) and isobologram (**right**), indicating synergistic, additive and antagonistic combinations. Right: Dose-response plot of two-drug checkerboard assay (3D plot), with drug-drug interactions mapped over it (color of the areas).

Bliss scoring

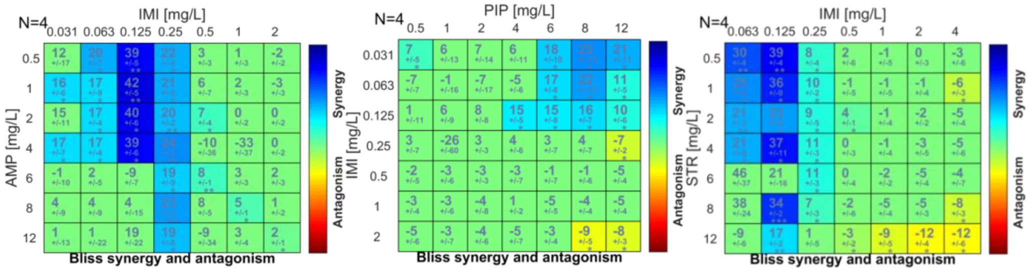

Figure S6. Synergy scores for drug-drug combinations containing imipenem for the strain producing beta-lactamase. Statistical significance: * $P < 5 \times 10^{-2}$, ** $< 10^{-3}$, *** $P < 10^{-4}$. Green-colored areas represent statistically insignificant values.

Readout Using pH Indicator

Readout of the mAST can be performed in more than one way. In Figure S7 we show that we can use phenol red, pH indicator, as the proliferation indication of the *E. coli*. Glucose from the medium is eaten by the proliferating cells, which convert the sugar into carbon dioxide (CO_2). CO_2 dissolves in the medium and acidifies the environment, causing change of phenol red hue from red to yellow. It would be also possible to use other dye, such as resazurin/resorufin or dodecylresorufin [2] and read the results by cell phone or confocal microscope. It was previously reported that leakage of fluorescent molecules may occur due to micelle mediated molecular transport [3]. This effect is however related to the presence of the perfluoropolyether-polyethylenoxide block copolymer (PFPE–PEG–PFPE) surfactant in fluorocarbon oils. In our experiments we didn't use any surfactants and in the chips cultured overnight (Figure S7 right) we didn't see any leakage, suggesting that in a surfactant-free system leakage is absent or negligible. Although antibiotics chemically differ from the pH indicator, the sole possibility that such leakage may theoretically occur was one of the reasons we decided for the OD detection method instead of a pH indicator.

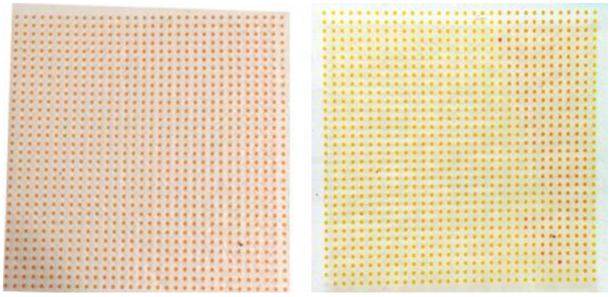

Figure S7. mAST readout using pH indicator. **Left**: mAST spotted with ampicillin (horizontally) and clindamycin (vertically), final concentration of each drug in well 0–256 μg/mL. **Right**: the same chip after overnight incubation.

Dose-Response Plots

Drugs of various concentrations were spotted into the microwells of the mAST device. The device was then filled with bacteria, and wells were separated by oil, as explained in Materials and Methods. Readout was performed as described in Materials and Methods. Each experiment was carried out in 4 repetitions. Dose-response plots were plotted in COMBENEFIT. The software automatically calculates EC_{50} value (measure of drug potency, for which concentration the response is equal to half of the response from control) or, should it be impossible, EC_{95} (analogically, for which drug concentration the response is equal to 95% of the control).

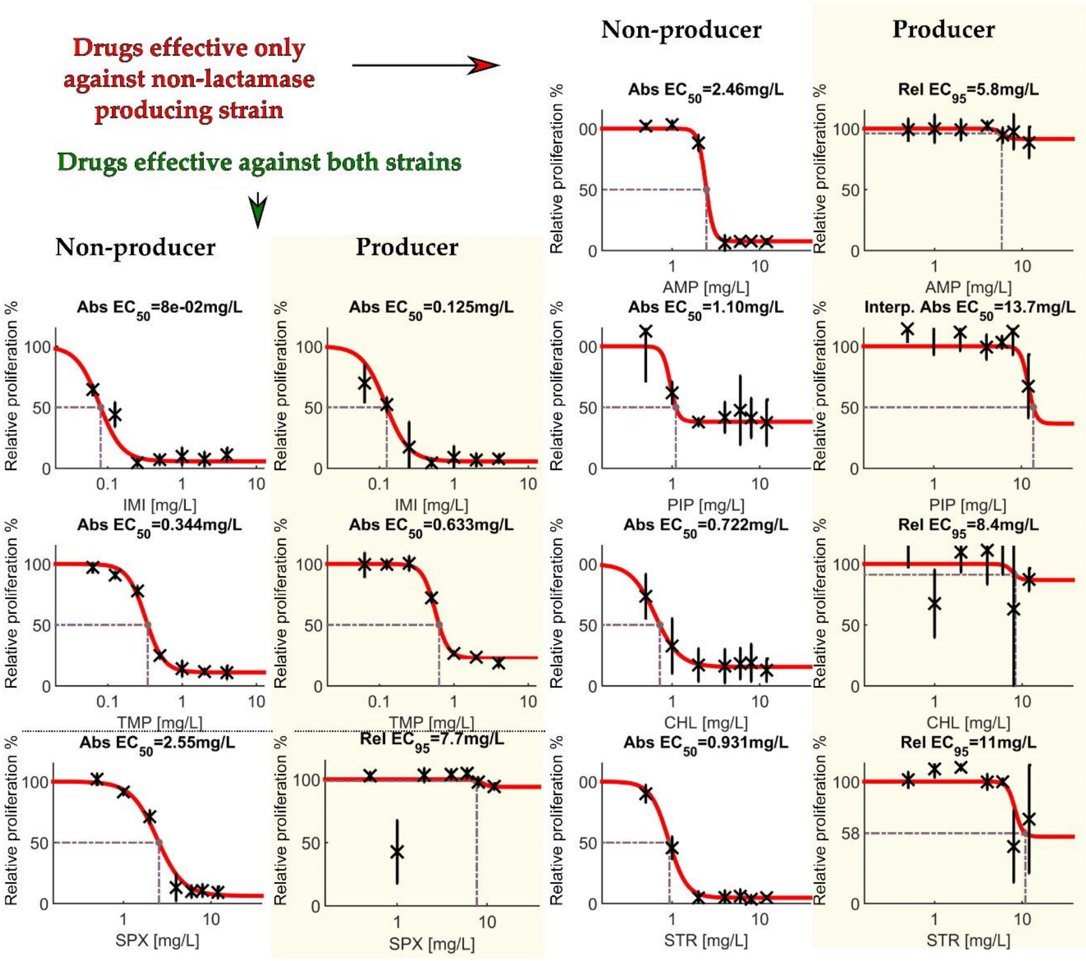

Figure S8. Dose-response plots of drugs against reference and resistant strains. Results for resistant (β-lactamase producer) strain are marked with yellow background **Left**: Drugs effective against both strains. **Right**: Drugs effective against β-lactamase non-producing reference strain only.

Variability of Readout Signal

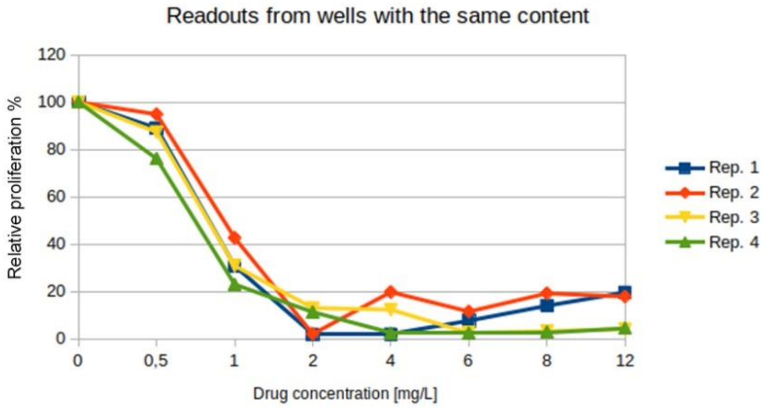

Figure S9. Example of readout variability of a drug dose-response test, ran in 4 repetitions simultaneously. The used drug is streptomycin, the used strain is beta-lactamase non-producing *E. coli*.

References

1. Di Veroli, G.Y.; Fornari, C.; Wang, D.; Mollard, S.; Bramhall, J.L.; Richards, F.M.; Jodrell, D.I. Combenefit: An interactive platform for the analysis and visualization of drug combinations. *Bioinformatics* **2016**, *32*, 2866–2868.
2. Scheler, O.; Kaminski, T.S.; Ruszczak, A.; Garstecki, P. Dodecylresorufin (C12R) Outperforms Resorufin in Microdroplet Bacterial Assays. *ACS Appl. Mater. Interfaces* **2016**, *8*, 11318–11325.
3. Gruner, P.; Riechers, B.; Semin, B.; Lim, J.; Johnston, A.; Short, K.; Baret, J.C. Controlling molecular transport in minimal emulsions. *Nature Communicaions* **2016**, 7, Article Number: 10392

© 2019 by the authors. Submitted for possible open access publication under the terms and conditions of the Creative Commons Attribution (CC BY) license (http://creativecommons.org/licenses/by/4.0/).
